# Whole-Body Electromyostimulation Combined With Individualized Nutritional Support Improves Body Composition in Patients With Hematological Malignancies – A Pilot Study

**DOI:** 10.3389/fphys.2018.01808

**Published:** 2018-12-18

**Authors:** Kristin Schink, Dejan Reljic, Hans J. Herrmann, Julia Meyer, Andreas Mackensen, Markus F. Neurath, Yurdagül Zopf

**Affiliations:** ^1^Hector-Center for Nutrition, Exercise and Sports, Department of Medicine 1, University Hospital Erlangen, Friedrich–Alexander University Erlangen–Nürnberg, Erlangen, Germany; ^2^Department of Medicine 5 – Haematology and Oncology, University Hospital Erlangen, Friedrich–Alexander University Erlangen–Nürnberg, Erlangen, Germany; ^3^Department of Medicine 1 – Gastroenterology, Pneumology and Endocrinology, University Hospital Erlangen, Friedrich–Alexander University Erlangen–Nürnberg, Erlangen, Germany

**Keywords:** body composition, physical function, whole-body electromyostimulation, exercise, nutrition, skeletal muscle mass, hematological malignancies, cancer

## Abstract

Patients undergoing the complex treatment for hematological malignancies are exposed to a high physiological and psychological distress inducing fatigue and physical inactivity. In line with cancer-related metabolic changes patients are predisposed for skeletal muscle mass loss that leads to a functional decline, affects therapeutic success, and quality of life. Benefits of physical exercise and nutritional interventions on muscle maintenance are observed in solid cancer patients, but marginally investigated in patients with hematological cancer. We here studied the effects of a combined supportive exercise and nutrition intervention using whole-body electromyostimulation (WB-EMS) training and individualized nutritional support in patients actively treated for hematological malignancy. In a controlled pilot trial, 31 patients (67.7% male; 58.0 ± 16.7 years) with various hematological cancers were allocated to a control group (*n* = 9) receiving nutritional support of usual care regarding a high protein intake (>1.0 g/kg/d) or to a physical exercise group (*n* = 22) additionally performing WB-EMS training twice weekly for 12 weeks. Bodyweight and body composition assessed by bioelectrical impedance analysis were measured every 4 weeks. Physical function, blood parameters, quality of life and fatigue were assessed at baseline and after 12 weeks. No WB-EMS-related adverse effects occurred. Patients attending the exercise program presented a higher skeletal muscle mass than controls after 12-weeks (1.51 kg [0.41, 2.60]; *p* = 0.008). In contrast, patients of the control group showed a higher fat mass percentage than patients of the WB-EMS group (-4.46% [-7.15, -1.77]; *p* = 0.001) that was accompanied by an increase in serum triglycerides in contrast to a decrease in the WB-EMS group (change ± SD, control 36.3 ± 50.6 mg/dl; WB-EMS -31.8 ± 68.7 mg/dl; *p* = 0.064). No significant group differences for lower limb strength, quality of life, and fatigue were detected. However, compared to controls the WB-EMS group significantly improved in physical functioning indicated by a higher increase in the 6-min-walking distance (*p* = 0.046). A combined therapeutic intervention of WB-EMS and protein-rich nutritional support seems to be safe and effective in improving skeletal muscle mass and body composition in hematological cancer patients during active oncological treatment.

**Clinical Trial Registration:** www.ClinicalTrials.gov, identifier NCT02293239.

## Introduction

With an increasing trend, 40,000 newly diagnosed cases of malign hematological diseases were registered and accounted for approximately 19,000 deaths in Germany in 2013 (Robert-Koch-Institut, 2016). The main malignant neoplasms comprise leukemia, Hodgkin and Non-Hodgkin lymphoma and multiple myeloma. Treatment regimens for affected patients are complex involving high-dose chemotherapy and total-body irradiation probably before or followed by hematopoietic stem cell transplantation. Even though improved therapy modalities had increased the survival of the patients in the past years, adverse therapy effects are still responsible for many deaths in hematological patients (Copelan, 2006; Othus et al., 2014; Buckley et al., 2015). Side effects linked to the disease as well as therapy complications such as anemia, a high susceptibility for infections, fatigue or Graft-versus-Host-Disease (GvHD) lead to a decreased physical activity – especially in patients with a long inpatient treatment (Danaher et al., 2006; Morishita et al., 2012a; Wiskemann et al., 2014). As a consequence of physical inactivity, the catabolic effects of cytotoxic and immunosuppressive therapies, as well as the metabolic changes and myopathy induced by a long-term glucocorticoid treatment (Gupta and Gupta, 2013; Macedo et al., 2016), hematological cancer patients undergo unfavorable body composition changes during the active treatment period (Greenfield et al., 2014). While fat mass increases during and after the oncological therapy, muscle mass declines. Those alterations are hardly reversible into the pre-illness and pre-treatment status and may result in sarcopenic obesity that may predispose patients for other metabolic diseases in future (Morishita et al., 2012b; Orgel et al., 2016; Xiao et al., 2016). Treatment-associated muscle weakness also leads to functional declines that can be still observed in survivorship (Inaba et al., 2012; Hung et al., 2013). Muscle wasting and physical deconditioning thereby not only enhance symptoms of fatigue, but also substantially impair patients’ quality of life, reduce therapy options and worsen prognosis (Danaher et al., 2006; Mosher et al., 2009; Morishita et al., 2012a,b; Lanic et al., 2014; Chu et al., 2017).

Adverse disease and treatment effects such as nausea, vomiting, diarrhea and mucositis can impair nutritional intake and are responsible for the high numbers of hematological patients that are at nutritional risk – particularly in patients who received stem cell transplantation (Liu et al., 2016). Thus, an adequate individual nutritional support is highly emphasized for hematological cancer patients as malnutrition could increase mortality (Calleja Fernandez et al., 2015).

Previous studies clearly suggest the feasibility of exercise intervention in hematological cancer patients to stabilize muscular status, body composition and improve physical activity (Liu et al., 2009). However, most studies were conducted as post-treatment rehabilitation interventions. A primary investigation of clinically relevant outcomes such as changes in skeletal muscle mass during treatment has been neglected so far. Treatment-related effects including fatigue and physical discomfort, but also bone lesions as a complication of multiple myeloma, may hinder actively treated patients to enter time-consuming and strenuous physical exercise programs. We therefore tested the feasibility of the innovative strength training method of whole-body electromyostimulation (WB-EMS) in hematological cancer patients undergoing treatment. The WB-EMS technology enables a simultaneous muscle contraction of all large muscle groups that is additionally supported by easy-to-perform dynamic exercises, as we described in detail previously (Schink et al., 2018). Observed benefits of a dual intervention of WB-EMS and individualized nutritional support on muscle mass in patients with advanced solid cancers (Schink et al., 2018), led us to the primary hypothesis that this combined approach is also feasible in hematological patients and may show better effects on stabilizing or even increasing skeletal muscle mass than a dietary support alone. Within this multimodal approach we also investigated the effect of the exercise and nutrition therapy on objective outcomes including bodyweight and body composition, physical functioning, hematological and blood chemistry parameters as well as on patient-reported quality of life and fatigue.

## Materials and Methods

### Patients

Adult patients (≥18 years) with confirmed diagnosis of a malignant hematological disease undergoing active treatment at initial evaluation were considered eligible for the study. This included patients with or without prior or subsequent hematopoietic stem cell transplantation (HSCT) and patients who were treated for acute GvHD as an adverse effect of HSCT. To enable the conduction of exercises patients had to display a Karnofsky performance status ≥60 at baseline. Patients were excluded from study participation when they already participated in other nutritional or physical exercise trials and consumed anabolic drugs, recently. An study exclusion was also necessary if patients experienced acute cardio-vascular events, suffered from epilepsy, severe neurological diseases or skin lesions within the area of electrodes, underwent surgery in the last 3 months, presented acute vein thrombosis, a cardiac pace-maker or conductive implants or in case of pregnancy as those conditions would exclude a WB-EMS application and bioelectrical impedance analysis measurement (Schink et al., 2018).

The declaration of the written informed consent to participate was obtained from every patient before his inclusion into the study. The study was conducted according to the guidelines of the Declaration of Helsinki. The protocol of this study was approved by the ethics committee of the Friedrich–Alexander University Erlangen–Nürnberg (Reg.Nr.155_13B) and registered at clinicaltrials.gov (NCT02293239). Participants of the present trial were recruited from the Department of Medicine 5 – Haematology and Oncology at the University Hospital Erlangen during November 2013 and March 2017. All baseline and outcome assessments, the WB-EMS exercise program, dietary counseling and data collection were performed at the Department of Medicine 1 – Gastroenterology, Pneumology and Endocrinology, Hector-Center for Nutrition, Exercise and Sports at the University Hospital Erlangen.

### Study Design

The pilot study was conducted as a two-armed prospective controlled clinical trial. After baseline assessment patients were either allocated to a physical exercise group regularly performing a WB-EMS training (WB-EMS group) or to a control group without any exercise intervention. Both groups received the same nutritional support during the study. Allocation to the study group was limited by the patients’ ability to attend the exercise program twice weekly, as described previously (Schink et al., 2018). In case of inability due to logistical aspects, patients were allocated to the control group resulting in an approximately 2:1 partition favoring the WB-EMS group. For each patient the duration of the study intervention was 12 weeks. Patients and outcome assessing personnel were not blinded.

### Dietary Support

The nutritional risk of the recruited study patients was assessed at baseline and documented by the nutritional risk screening (NRS-2002) (Kondrup et al., 2003). Patients who scored greater or equal three points were assumed to be at nutritional risk caused by low body mass index, decreased food intake and/or prior involuntary weight loss (Kondrup et al., 2003). The dietary support and monitoring of the nutritional intake by weekly 24 h-dietary records (Freiburger Ernährungsprotokoll; Nutri-Science GmbH, Freiburg) during the study have been described previously (Schink et al., 2018). Briefly, dietary intake was analyzed by Prodi^®^6 (Nutri-Science GmbH, Freiburg) and a certified dietician instructed/motivated patients by face-to-face conversation to achieve a daily protein intake of >1.0 g/kg and caloric intake of at least 25 kcal/kg bodyweight in regard to current dietary recommendations for patients with malignant diseases (Arends et al., 2015, 2016). As a precautionary measure, patients suffering from renal insufficiency or displaying high serum creatinine concentrations (women, >1.0 mg/dl; men, >1.2 mg/dl) were instructed not to exceed a daily protein intake of 1.0 g/kg in acute or 1.2 g/kg in chronic renal disease (Arends et al., 2016). Supplemental nutrition in form of protein/amino acid-rich oral supplements or parenteral nutrition (Olimel 5.7% E, Baxter Germany, Munich) was provided to patients with insufficient or impaired nutrient intake.

To assess the compliance to the dietary recommendations proportions of patients who achieved only a normal (<25 kcal/kg/d; ≤1.0 g/kg/d) or an increased (≥25 kcal/kg/d; >1.0 g/kg/d) protein and energy intake were calculated.

### Physical Exercise Program

Study intervention included a physical exercise program in the form of a regular WB-EMS training over a period of 12 weeks. The conduction of the WB-EMS training and exercises were detailed described previously (Schink et al., 2018). Electrical muscle stimulation was applied by bipolar impulses at a frequency of 85 Hz and pulse width of 350 μs mediating a stimulation for 6 s followed by a 4 s stimulation rest. Here, muscles of the upper legs, upper arms, bottom, abdomen, chest, lower back, upper back, and latissimus dorsi have been stimulated. The supervised WB-EMS training was conducted twice a week for 20 min/session and comprised a sequence of light dynamic and easy-to-perform physical exercises that supported the activation of the mentioned muscle groups (Schink et al., 2018). WB-EMS equipment was purchased from Miha bodytec (Miha bodytec GmbH, Gersthofen).

### Study Outcomes

#### Assessments

Demographic data were collected by an anamnesis questionnaire and medical records. Assessments of study outcomes on body composition were performed at baseline, in week 4, week 8 and at study end after 12 weeks of study intervention. Blood parameters, physical functioning and patient-reported quality of life and fatigue were assessed at baseline and study end.

The adherence of the patients to the WB-EMS training was registered by the supervising physiotherapists at each exercise session. Exercise adherence rate of the patients who completed the 12-week period of the study was calculated by the number of performed trainings from a total of 24 WB-EMS trainings.

#### Body Composition, Physical Function, and Quality of Life

The primary outcome was the change of skeletal muscle mass assessed by bioelectrical impedance analysis – BIA (seca mBCA 515; Seca GmbH & Co., KG, Hamburg) (Bosy-Westphal and Jensen, 2017). Secondary endpoints of bodyweight and body composition including fat mass percentage and phase angle were also assessed by BIA.

Physical functioning and endurance were assessed by the 6-min-walk test (Schmidt et al., 2013). Functioning and strength of the lower limbs was indirectly determined by the 30 s sit-to-stand (STS) test (Jones et al., 1999). Physical performance status was determined by Karnofsky-Index (Karnofsky and Burchenal, 1949).

Patient-reported quality of life was evaluated by the European Organisation for Research and Treatment of Cancer Quality of Life Questionnaire – C30 (EORTC QLQ-C30) (Aaronson et al., 1993). Here, a higher score in global health and functional scales indicate a better quality of life while high scores in symptom scales hint toward a higher symptomatic burden. An additional assessment of fatigue was done by the use of the Functional Assessment of Chronic Illness Therapy – Fatigue Scale that indicates less fatigue and better function by a higher score (13-item FACIT Fatigue Scale) (Lai et al., 2003).

#### Analysis of Blood Samples

Blood samples were collected at baseline and after 12 weeks of intervention by puncture of the arm vein or blood values were extracted from the documented routine blood sampling undertaken during the oncological treatment. The analysis of nutritional and inflammatory blood markers (normal values: C-reactive protein, CRP <5 mg/l; albumin 35–55 g/l; total protein 66–83 g/l; triglycerides 50–200 mg/dl; creatinine 0.51–1.17 mg/dl; lactate dehydrogenase, LDH <250 U/l) and hematological parameters (leukocytes 4.4–11.3 × 10^3^/μl, thrombocytes 150–300 × 10^3^/μl, hematocrit 35–48%, hemoglobin 11.5–18.0 g/dl, erythrocytes 4.1–6.0 × 10^6^/μl) was carried out by the diagnostic laboratories of the University Hospital Erlangen.

### Statistical Analysis

Descriptive data are presented as means ± standard deviations (SD) for continuous variables and count and percentages for categorical variables. Laboratory variables are expressed as median with range. Normal distribution of data was tested by Shapiro–Wilk test. Baseline differences between study groups were analyzed by Pearson’s chi-squared test and Fisher’s exact test, where appropriate, for categorical variables. Continuous variables were analyzed by independent-samples *t*-test, Welch-test or Mann–Whitney *U*-test, where appropriate. Similarly, comparisons between patients who completed and not completed the study were conducted.

To evaluate the effect of WB-EMS intervention on skeletal muscle mass, bodyweight, and body composition throughout the study course, linear mixed models (LMM) were generated. LMM allow the analysis of longitudinal data with missing samples that is common for clinical trials including serious ill patients with expected high dropout rates and thus follows the intent-to-treat approach (Chakraborty and Gu, 2009; West, 2009; Peters et al., 2012). The LMM were fitted for each outcome using a patient-specific random intercept. To control for baseline differences, the baseline value of each outcome was included in the models. Time and a time-group interaction to estimate the intervention effect on the study outcome over time were included as fixed effects. As part of a sensitivity analysis, we also assessed fixed effects adjusted for age, gender, diagnosis of the hematological malignancy and glucocorticoid intake. Results are presented as parameter estimates with 95% confidence intervals (95% CI). To assess an association between protein intake and changes in skeletal muscle mass a Pearson correlation analysis was carried out for both study groups with patients presenting skeletal muscle mass data after 12 weeks. Secondary outcomes only assessed at baseline and after 12 weeks were analyzed for all patients with pre- and post-intervention data. Pre- to post-test changes were compared by the use of independent-samples *t*-test or Mann–Whitney *U*-test, where appropriate, and presented as mean ± SD.

Statistical analysis was conducted using SPSS version 21 (IBM SPSS Statistics, Ehningen, Germany) and Prism 7.00 (GraphPad Software Inc., La Jolla, CA, United States). Two-sided *p*-value <0.05 was considered as statistically significant. Due to the exploratory character of this pilot study, no correction for multiple testing was applied.

## Results

### Participants

#### Baseline Characteristics

**FIGURE 1:**
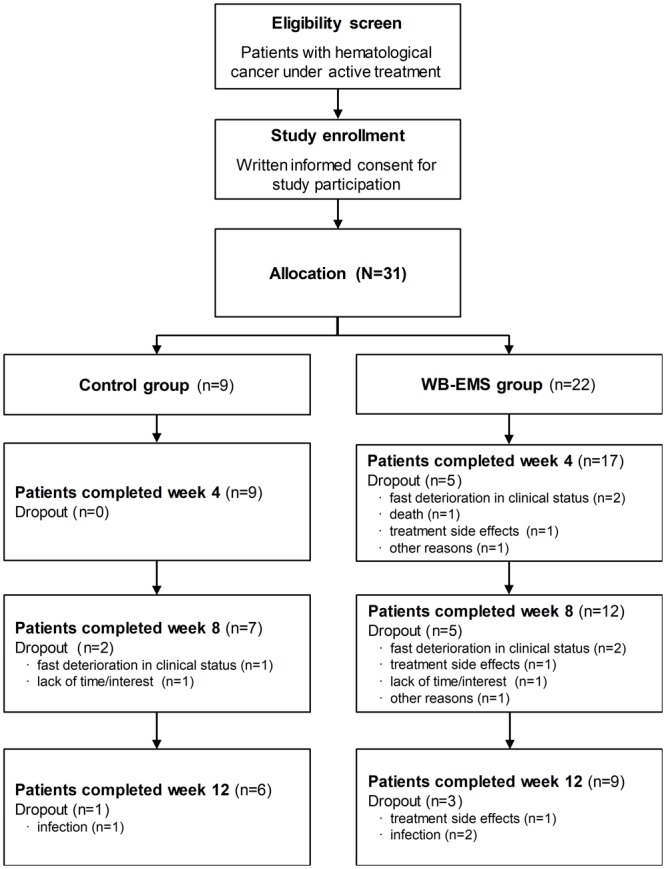
Patient flowchart. The flowchart shows the number of the allocated patients and the number of patients who dropped out during study course with dropout reasons and the number of patients who completed the whole intervention period of 12 weeks. During the study, body composition analysis was missed by 1 patient of the WB-EMS group at week 4 and 1 control and 2 WB-EMS patients at week 8. WB-EMS, whole-body electromyostimulation.

Thirty-one patients undergoing active treatment for acute myeloid leukemia (AML), Hodgkin lymphoma, Non-Hodgkin lymphoma (NHL), multiple myeloma and/or associated acute GvHD were recruited for the trial (Figure 1).

Participants’ baseline characteristics are presented in Table 1. Study groups were well balanced in demographic and anthropometric baseline variables with a higher percentage of male participants in both study groups. No significant group differences between body composition parameters and functional status were observed at study entry. Patients of both groups showed a high mean body mass index (>25.0 kg/m^2^) and 61.3% of all recruited patients were defined as overweight or obese regarding the WHO classification of the nutritional status (World Health Organization [WHO], 2000). The obese phenotype of the patients was underlined by a high fat mass percentage (>27.0% fat mass) and increased metabolic risk factors indicated by higher serum triglyceride concentrations (>170 mg/dl). Nevertheless, approximately two-third of the study patients in both groups were at nutritional risk (NRS score ≥3) due to decreased food intake or unintended disease- or treatment-related weight loss indicating the demand of nutritional support at baseline. However, only one patient of the control and three patients of the WB-EMS group had to be supported by artificial nutrition in addition to the support by nutritional counseling. Patients of both study groups reported comparable quality of life (*p* = 0.286) and fatigue severity (*p* = 0.284) at the beginning of the study. Disease characteristics including diagnosis and current oncological treatment of the hematological cancer were not significantly different at baseline except of a significantly lower median leukocyte count in the control group compared to the WB-EMS group. Approximately one-third of the study patients of each group exhibited osteolytic lesions as a consequence of myeloma bone marrow infiltration.

**Table 1:** Demographic and disease characteristics of the included patients.

	Control	WB-EMS	
Parameter	(*n* = 9)	(*n* = 22)	*p*-Value^a^
Gender			1.000
Male	6 (66.7)	15 (68.2)	
Female	3 (33.3)	7 (31.8)	
Mean age	57.2 ± 14.1	54.8 ± 17.9	0.881
**Functional status**			
Karnofsky-Index	73.3 ± 8.7	75.9 ± 11.4	0.548
6-min walking distance (m)	504.7 ± 66.1	494.0 ± 142.2	0.832
Sit-to-stand repetitions	12.7 ± 3.7	12.6 ± 5.1	0.953
**Anthropometry**			
BMI [kg/m^2^]^2^	25.8 ± 3.1	25.4 ± 4.1	0.810
Underweight (<18.5 kg/m^2^)	0 (0.0)	2 (9.1)	0.479
Normal weight (18.5–24.9 kg/m^2^)	4 (44.4)	6 (27.3)	
Overweight/Obesity (≥25.0 kg/m^2^)	5 (55.6)	14 (63.6)	
Fat mass index (kg/m^2^)	7.1 ± 2.1	7.4 ± 3.1	0.829
Fat free mass index [kg/m^2^]	18.6 ± 2.1	18.3 ± 2.4	0.741
**Body composition**			
Skeletal muscle mass [kg]	25.7 ± 6.6	24.7 ± 6.3	0.704
Fat mass [%]	27.4 ± 6.1	27.6 ± 9.7	0.578
Hydration [%; ECW:ICW]	87.6 ± 15.1	86.6 ± 10.3	0.823
Phase angle [°]	4.4 ± 1.1	4.2 ± 0.9	0.625
**Nutritional status**			
Nutritional risk screening			1.000
(NRS-2002)
<3	3 (33.3)	8 (36.4)	
≥3	6 (66.7)	14 (63.6)	
Weight loss [%]	5.2 ± 4.0	4.3 ± 4.5	0.334
≤5%	4 (44.4)	16 (72.7)	0.217
>5%	5 (55.6)	6 (27.3)	
Nutritional therapy			1.000
Only dietary counseling	8 (88.9)	19 (86.4)	
Oral supplementation	1 (11.1)	3 (13.6)	
Parenteral nutrition	0 (0.0)	0 (0.0)	
**Quality of life**			
EORTC QLQ-C30 global	45.3 ± 16.5	54.5 ± 20.0	0.286
health
FACIT-fatigue scale	27.3 ± 10.4	32.6 ± 11.1	0.284
**Disease characteristics**			
Diagnosis			0.272
Acute myeloid leukemia	3 (33.3)	2 (9.1)	
Hodgkin lymphoma	2 (22.2)	3 (13.6)	
Non-Hodgkin-lymphoma	1 (11.1)	7 (31.8)	
Multiple myeloma	3 (33.3)	10 (45.5)	
Oncological treatment^1^			
Chemotherapy	7 (77.8)	9 (40.9)	0.113
Radiotherapy	1 (11.1)	2 (9.1)	1.000
Targeted/immunotherapy	6 (66.7)	15 (68.2)	1.000
Glucocorticoid intake	6 (66.7)	17 (77.3)	0.660
Immunosuppression	5 (55.6)	15 (68.2)	0.683
Previous HSCT	1 (11.1)	6 (27.3)	0.639
Osteolytic lesion	3 (33.3)	7 (31.8)	1.000
Number of medications	5.9 ± 3.6	7.2 ± 6.0	0.881
Comorbidities^1^			
Cardiovascular disease	3 (33.3)	6 (27.3)	1.000
Pulmonary disease	0 (0.0)	2 (9.1)	1.000
Liver disease	1 (11.1)	1 (4.5)	0.503
Renal failure	2 (22.2)	1 (4.5)	0.195
Thyroid disease	2 (22.2)	4 (18.2)	1.000
Diabetes mellitus	0 (0.0)	1 (4.5)	1.000
Hypertension	4 (44.4)	6 (27.3)	0.417
Hyperlipidemia	1 (11.1)	3 (13.6)	1.000
Acute GvHD	0 (0.0)	2 (9.1)	1.000
**Laboratory values**			
Leukocytes [×10^3^/μl]	3.60 (1.30–11.40)	5.45 (2.10–27.90)	**0.016**
Thrombocytes [×10^3^/μl]	137.0 (56.0–367.0)	183.5 (26.0–383.0)	0.676
Erythrocytes [×10^6^/μl]	3.77 (2.93–4.97)	3.77 (2.71–4.85)	0.996
Hematocrit [%]	32.9 (26.9–41.7)	34.1 (27.8–43.3)	0.607
Hemoglobin [g/dl]	12.1 (9.2–13.9)	11.8 (8.7–14.3)	0.769
Albumin [g/l]	40.6 (35.5–43.6)	39.1 (24.7–46.2)	0.290
C-reactive protein [mg/l]	1.9 (0.6–16.7)	3.4 (0.2–82.9)	0.749
Creatinine [mg/dl]	0.94 (0.60–1.77)	0.86 (0.61–3.11)	0.334
Lactate dehydrogenase	220.0 (48.0–1101.0)	233.5 (103.0–638.0)	0.593
[IU/l]
Triglycerides [mg/dl]	174.0 (51.0–695.0)	187.0 (79.0–319.0)	0.377

*Data are presented in numbers and proportions (%) and as mean ± standard deviation. Laboratory values are expressed as median and range (min to max). ^*a*^Baseline comparison between WB-EMS and control group assessed by Pearson’s chi-squared and Fisher’s exact test, respectively, for categorical variables and independent-samples *t*-test, Welch’s test or Mann–Whitney *U*-test for continuous variables. Statistically significant differences are indicated by *p* < 0.05 and marked in bold type. ^*1*^Numbers of patients for comorbidities and oncological treatment include also patients with several comorbidities and combined anticancer therapies. ^*2*^WHO categories to determine nutritional status (World Health Organization [WHO], 2000). WB-EMS, whole-body electromyostimulation; BMI, body mass index; ECW, extracellular water; ICW, intracellular water; GvHD, Graft-versus-Host-Disease; HSCT, hematopoietic stem cell transplantation.*

#### Nutritional Intake

During the study the nutritional goals regarding a high daily caloric (≥25 kcal/kg) and protein (>1.0 g/kg) intake were achieved by approximately 90% of all recruited patients of the control and the WB-EMS group (Table 2). Both study groups exceeded the minimum aimed dietary intake by a mean daily protein intake of 1.4 g/kg and a mean daily caloric intake more than 30 kcal/kg. Of note, two patients of the WB-EMS group and one control patient failed to document dietary intake and were therefore unavailable for the nutritional analysis.

**Table 2:** Nutritional intake during study course of the included patients^a^.

	Study group	
	Control	WB-EMS	
	(*n* = 8)	(*n* = 20)	*p*-Value^b^
**Mean daily nutrient/caloric**			
**intake [per kg bodyweight]**
Carbohydrates (g)	3.4 ± 0.8	3.6 ± 1.2	0.650
Fat (g)	1.4 ± 0.5	1.4 ± 0.5	0.636
Protein (g)	1.4 ± 0.3	1.4 ± 0.2	0.733
Energy (kcal)	32.8 ± 8.7	34.2 ± 7.5	0.669
**Achievement of dietary**			
**goals**
Energy group			1.000
<25 kcal/kg	1 (12.5)	2 (10.0)	
≥25 kcal/kg	7 (87.5)	18 (90.0)	
Protein group			1.000
≤1.0 g/kg	1 (12.5)	2 (10.0)	
>1.0 g/kg	7 (87.5)	18 (90.0)	

*Data are presented in numbers and proportions (%) and as mean ± standard deviation. ^*a*^Dietary data of two patients of the WB-EMS group and one patient of the control group were unavailable. ^*b*^Categorical variables were analyzed by Fisher’s exact test and continuous variables by independent-samples *t*-test. WB-EMS, whole-body electromyostimulation.*

### Feasibility and Exercise Adherence

Patients’ flow chart and dropout reasons are presented in Figure 1. During the trial, 3 patients of the control and 13 patients of the WB-EMS group prematurely withdrew from the study leading to a dropout rate of 33.3% for the control and 59.1% for the WB-EMS group (*p* = 0.252). Reason for study dropout in the WB-EMS group was mainly due to treatment-related toxicities and clinical deterioration. Two patients of the WB-EMS group who prematurely terminated the study suffered from acute GvHD. However, reasons were not significantly different between study groups (*p* = 0.686). Patients who were not able to continue the study had a significantly higher drug intake (8.9 ± 6.0 vs. 4.7 ± 3.7; *p* = 0.026) as well as a higher leukocyte count (9.0 ± 7.2 × 10^3^/μl vs. 4.6 ± 2.4 × 10^3^/μl; *p* = 0.033) in line with lower albumin concentrations (37.7 ± 5.1 g/l vs. 40.7 ± 3.5 g/l; *p* = 0.073) than patients who completed the study. Further, they showed a less social functioning assessed by the EORTC QLQ-C30 (44.9 ± 19.8 vs. 65.5 ± 21.2; *p* = 0.015). No significant differences of the patients who dropped out between the two study groups were detected. Remarkably, no WB-EMS related side-effects or discomfort appeared despite a small muscular aching comparable to other strength training methods. Patients of the WB-EMS group who completed the 12-week intervention period attended in average 18.6 ± 3.5 from a total of 24 training sessions leading to a mean exercise adherence rate of 77.3 ± 14.5%.

### Body Composition

**FIGURE 2:**
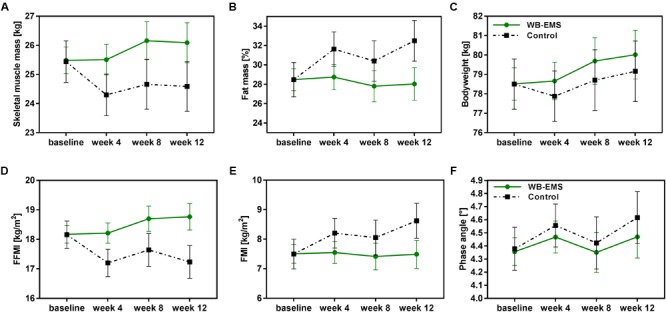
Changes in body composition during the 12-week intervention period. Unadjusted estimated marginal means with 95% confidence intervals of the body composition parameters **(A)** skeletal muscle mass, **(B)** fat mass percentage, **(C)** bodyweight, **(D)** FFMI, **(E)** FMI, and **(F)** phase angle are illustrated for the control group and the WB-EMS group over the 12-week study course. WB-EMS, whole-body electromyostimulation; FMI, fat mass index; FFMI, fat free mass index.

During the study course of 12 weeks patients of the WB-EMS group showed an increase in skeletal muscle mass, while a reduction in muscle mass was observed for control patients (Figure 2A). This resulted in a significant estimated mean difference between the groups of 1.21 kg [0.32, 2.10] at week 4, 1.49 kg [0.41, 2.57] at week 8, and 1.51 kg [0.41, 2.60] at the end of the 12-week intervention period favoring the combined exercise and nutrition intervention (*p* < 0.01; Table 3). In line with this, fat free mass index (FFMI) was significantly higher in the WB-EMS group compared to controls after 12 weeks (*p* < 0.001; Figure 2D and Table 3). Both study groups tended to increase in bodyweight (Figure 2C), but in contrast to the WB-EMS group, the bodyweight gain in controls was characterized by an increase in fat mass indicated by an increase of the body fat percentage (Figure 2B) and fat mass index (FMI; Figure 2E). This led to an estimated negative effect of the WB-EMS group on body fat percentage and FMI (*p* < 0.01 at week 12; Table 3). No significant mean group differences were observed for the parameter of phase angle over time (*p* > 0.05; Table 3). A trend toward an increased phase angle in controls (Figure 2F) is suggested to be rather a result of treatment-related changes in body water distribution of the extracellular water (ECW) to the intracellular water (ICW) (Hydration, Table 3) than in body cell mass. Of note, an additional sensitivity analysis adjusting the LMM’s for age, gender, cancer diagnosis, and glucocorticoid treatment did not reveal appreciable alterations of the estimated treatment effects and significances (Table 3).

**Table 3:** Linear mixed model analysis estimating the unadjusted and adjusted^a^ effect (group × time) of the combined WB-EMS and nutrition intervention on anthropometry, and body composition over the 12-week study course compared to the usual care control group.

		Estimated effect of WB-EMS intervention compared to controls^b^					
		Estimate [95% CI]	*p*-Value	Estimate [95% CI]	*p*-Value	Estimate [95% CI]	*p*-Value
		
Outcome measure	Model	Week 4		Week 8		Week 12	
**Anthropometry**							
Bodyweight [kg]	Unadjusted	0.78 [–0.83, 2.40]	0.336	0.99 [–0.98, 2.95]	0.321	0.85 [–1.15, 2.85]	0.399
	Adjusted**^a^**	0.80 [–0.80, 2.41]	0.320	1.08 [–0.88, 3.03]	0.276	0.94 [–1.04, 2.93]	0.347
FMI [kg/m^2^]	Unadjusted	–0.66 [–1.28, –0.04]	**0.039**	–0.64 [–1.39, 0.10]	0.091	–1.13 [–1.89, –0.38]	**0.004**
	Adjusted**^a^**	–0.75 [–1.42, –0.07]	**0.030**	–78 [–1.57, 0.01]	**0.054**	–1.21 [–2.02, –0.41]	**0.004**
FFMI [kg/m^2^]	Unadjusted	1.00 [0.43, 1.59]	**0.001**	1.06 [0.35, 1.76]	**0.004**	1.53 [0.82, 2.25]	**0.000**
	Adjusted**^a^**	1.10 [0.53, 1.66]	**0.000**	1.22 [0.53, 1.91]	**0.001**	1.70 [1.01, 2.41]	**0.000**
**Body composition**							
SMM [kg]	Unadjusted	1.21 [0.32, 2.10]	**0.008**	1.49 [0.41, 2.57]	**0.007**	1.51 [0.41, 2.60]	**0.008**
	Adjusted**^a^**	1.36 [0.48, 2.24]	**0.003**	1.75 [0.68, 2.81]	**0.002**	1.77 [0.68, 2.85]	**0.002**
FM [%]	Unadjusted	–2.89 [–5.08, –0.70]	**0.011**	–2.60 [–5.25, 0.05]	0.054	–4.46 [–7.15, –1.77]	**0.001**
	Adjusted**^a^**	–3.30 [–5.59, –1.01]	**0.006**	–3.27 [–6.00, 0.53]	**0.020**	–4.91 [–7.68, –2.13]	**0.001**
PhA [°]	Unadjusted	–0.09 [–.29, 0.12]	0.396	–0.07 [–0.32, 0.18]	0.572	–0.15 [–0.40, 0.11]	0.256
	Adjusted**^a^**	–0.10 [–0.32, 0.13]	0.389	–0.07 [–0.33, 0.20]	0.614	–0.14 [–0.41, 0.13]	0.294
Hydration [%, ECW: ICW]	Unadjusted	0.88 [–1.51, 3.26]	0.466	1.30 [–1.57, 4.17]	0.371	2.95 [0.02, 5.88]	**0.049**
	Adjusted**^a^**	0.94 [–1.37, 3.25]	0.419	1.06 [–1.74, 3.86]	0.454	2.93 [0.08, 5.78]	**0.044**

*^*a*^Models were adjusted for age, gender, diagnosis of hematological malignancy and glucocorticoid intake. ^*b*^Data are presented as estimated mean difference between study groups and 95% confidence intervals [95% CI]. Statistically significant effects are marked in bold type. WB-EMS, whole-body electromyostimulation; FMI, fat mass index; FFMI, fat free mass index; SMM, skeletal muscle mass, FM, fat mass; PhA, phase angle; ECW, extracellular water; ICW, intracellular water.*

**FIGURE 3:**
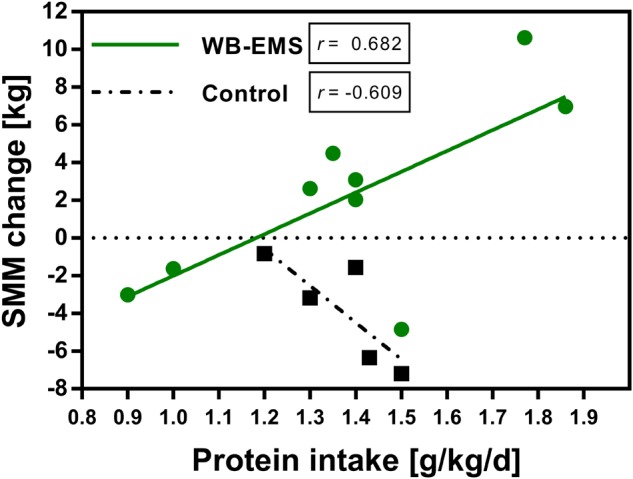
Relationship between daily protein intake and change in skeletal muscle mass. Pearson correlation between daily protein intake (g/kg bodyweight) and change in skeletal muscle mass (kg) of the control (*n* = 6) and the WB-EMS group (*n* = 9) after 12 weeks. SMM, skeletal muscle mass; WB-EMS, whole-body electromyostimulation.

Pearson correlation analysis yielded a significant strong positive correlation between an increase in skeletal muscle mass and the amount of protein intake in the WB-EMS group (*r* = 0.682; *p* = 0.043), while no significant relationship between protein intake and muscle mass gain was observed for the control group (*r* = -0.609; *p* = 0.275; Figure 3). The significant correlation for the WB-EMS group was also observed for muscle mass changes in week 4 and week 8 (*r* > 0.600), but not in the control group (data not shown).

### Physical Function and Quality of Life

Analysis of physical function, quality of life and fatigue were based on available pre- and post-assessment data measured at baseline and after 12 weeks (Table 4). Patients of the WB-EMS group significantly increased their 6-min-walking distance after 12 weeks (48.4 ± 51.5 m; *p* = 0.032), while walking performance was kept stable in controls, leading to a significant difference between the study groups (*p* = 0.046; Table 4). Though significance level between groups was not reached (*p* = 0.181), an increased performance status was reported by the patients of the WB-EMS group (Karnofsky-Index, 11.1 ± 17.6; *p* = 0.095). A comparable increase in patient-reported physical functioning was observed for both study groups (controls, 11.5 ± 18.3; WB-EMS group 10.8 ± 22.7; *p* = 0.957). Interestingly, controls reported a non-significant decline in social functioning after 12 weeks (-16.6 ± 27.3; *p* = 0.311), while exercising patients remained stable (2.0 ± 19.3; *p* = 0.764), and thus tended to function better socially (*p* = 0.183). Both study groups improved their fatigue symptoms (FACIT and C30 scale), whereby only the within-group difference of the controls for the FACIT-Fatigue scale reached statistical significance (11.3 ± 6.6; *p* = 0.042). However, no significant inter-group differences between changes in fatigue were revealed. Global health tended to improve within the WB-EMS group (10.4 ± 15.6; *p* = 0.080), but no significant differences between the groups were detected. Moreover, symptoms of insomnia significantly declined (-37.0 ± 35.2; *p* = 0.014) and nausea/vomiting tended to decrease in the WB-EMS group (-14.9 ± 22.8, *p* = 0.066).

**Table 4:** Physical function and quality of life at baseline and after 12 weeks^1^.

Outcome measure	Study group	*n*	Baseline	Week 12	Difference	*p*-Value^a^	*p*-Value^b^
**Physical function**							
SMWT distance [m]	Control	6	519.5 ± 66.7	512.1 ± 76.7	–7.4 ± 38.7	0.658	**0.046**
	WB-EMS	8	534.1 ± 76.0	582.6 ± 82.6	48.4 ± 51.5	**0.032**	
STS repetitions	Control	5	14.2 ± 3.0	13.4 ± 2.1	–0.8 ± 1.8	0.336	0.429
	WB-EMS	6	13.7 ± 2.7	13.5 ± 3.9	–0.2 ± 1.7	0.822	
Karnofsky-Index	Control	6	73.3 ± 10.3	75.0 ± 5.5	1.7 ± 7.5	0.611	0.181
	WB-EMS	9	75.6 ± 15.9	86.7 ± 14.1	11.1 ± 17.6	0.095	
**Quality of life**							
*FACIT-fatigue scale*	Control	4	25.5 ± 12.4	36.8 ± 8.8	11.3 ± 6.6	**0.042**	0.147
	WB-EMS	9	35.0 ± 12.5	39.8 ± 11.6	4.8 ± 7.0	0.075	
**EORTC QLQ-C30**							
*Functional scales*							
PF	Control	4	63.5 ± 32.7	75.0 ± 21.9	11.5 ± 18.3	0.299	0.957
	WB-EMS	9	66.0 ± 20.9	76.8 ± 23.4	10.8 ± 22.7	0.192	
RF	Control	4	60.8 ± 25.7	50.1 ± 19.5	–10.8 ± 18.3	0.570	0.261
	WB-EMS	9	49.9 ± 30.1	62.9 ± 20.1	13.0 ± 33.2	0.274	
EF	Control	4	66.6 ± 26.6	62.3 ± 24.8	–4.3 ± 33.8	0.818	0.710
	WB-EMS	9	64.8 ± 35.9	71.2 ± 23.4	6.4 ± 31.3	0.553	
CF	Control	4	60.4 ± 25.0	66.9 ± 0.2	6.5 ± 25.2	0.641	0.833
	WB-EMS	9	81.4 ± 21.1	90.8 ± 14.6	9.3 ± 20.5	0.208	
SF	Control	4	75.1 ± 9.3	58.5 ± 34.6	–16.6 ± 27.3	0.311	0.183
	WB-EMS	9	61.0 ± 25.1	63.0 ± 35.2	2.0 ± 19.3	0.764	
*Symptom scales*							
Pain	Control	4	29.0 ± 20.9	12.5 ± 15.4	–16.5 ± 13.5	0.092	0.928
	WB-EMS	9	44.4 ± 40.7	25.8 ± 35.3	–18.7 ± 45.2	0.250	
Dyspnea	Control	4	41.5 ± 42.0	41.5 ± 42.0	0.0 ± 0.0	1.000	0.598
	WB-EMS	9	36.9 ± 31.0	29.6 ± 42.3	–7.3 ± 40.1	0.598	
Insomnia	Control	4	58.3 ± 50.1	24.8 ± 16.5	–33.5 ± 38.7	0.157	0.940
	WB-EMS	9	48.1 ± 37.8	11.1 ± 23.6	–37.0 ± 35.2	**0.014**	
Appetite loss	Control	4	24.8 ± 16.5	16.5 ± 19.1	–8.3 ± 16.5	0.317	0.825
	WB-EMS	9	22.2 ± 29.0	18.4 ± 33.8	–3.8 ± 20.0	0.414	
Constipation	Control	4	33.3 ± 27.4	33.5 ± 38.7	0.3 ± 47.6	0.992	0.414
	WB-EMS	9	11.1 ± 23.6	3.7 ± 11.0	–7.4 ± 14.8	0.180	
Diarrhea	Control	4	8.3 ± 16.5	0.0 ± 0.0	–8.2 ± 16.5	0.317	0.825
	WB-EMS	9	11.1 ± 23.6	3.7 ± 11.0	–7.4 ± 27.8	0.414	
Financial difficulties	Control	4	16.8 ± 33.5	0.0 ± 0.0	–16.8 ± 33.5	0.317	0.503
	WB-EMS	9	14.8 ± 24.2	14.8 ± 24.2	0.0 ± 17.0	1.000	
Nausea/vomiting	Control	4	16.5 ± 19.1	8.3 ± 16.5	–8.3 ± 16.5	0.317	0.710
	WB-EMS	9	18.6 ± 25.7	3.7 ± 11.0	–14.9 ± 22.8	0.066	
Fatigue	Control	4	66.8 ± 24.1	44.3 ± 27.4	–22.5 ± 45.3	0.394	1.000
	WB-EMS	9	46.9 ± 23.0	36.9 ± 35.6	–10.0 ± 25.8	0.114	
Global Health	Control	4	45.8 ± 10.7	64.5 ± 14.2	18.8 ± 21.8	0.184	0.448
	WB-EMS	9	61.8 ± 24.0	72.2 ± 18.3	10.4 ± 15.6	0.080	

*^*1*^Includes patients with pre- and post-assessment data. ^*a*^Paired-sample *t*-test for parametric or Wilcoxon-signed-rank test for non-parametric values for comparison of pre- and post-intervention values. ^*b*^Independent-samples *t*-test for parametric or Mann–Whitney *U*-test for non-parametric values for comparison of pre- to post-test changes. Statistical significance is indicated by *p* < 0.05 and marked in bold type. SMWT, 6-min-walking test; STS, 30 s sit-to-stand test; PF, physical functioning; RF, role functioning; EF, emotional functioning; CF, cognitive functioning; SF, social functioning; WB-EMS, whole-body electromyostimulation.*

### Hematological and Biochemical Parameters

No significant within- and between-group differences were detected for hematological blood parameters (Table 5). However, a distinct decrease in triglyceride concentrations was noticed in the WB-EMS group (-31.8 ± 68.7; *p* = 0.232) in contrast to an increase in the control group (36.3 ± 50.6; *p* = 0.139), albeit differences between the groups did not reach level of statistical significance (*p* = 0.064).

**Table 5:** Hematological and blood biochemistry parameters at baseline and after 12 weeks^1^.

Outcome measure	Study group	*n*	Baseline	Week 12	Difference	*p*-Value^a^	*p*-Value^b^
**Hematological parameters**							
Leukocytes [×10^3^/μl]	Control	6	4.27 ± 3.63	3.86 ± 1.86	–0.41 ± 2.83	0.739	0.607
	WB-EMS	9	4.85 ± 1.44	4.68 ± 2.23	–0.17 ± 3.21	0.878	
Thrombocytes [×10^3^/μl]	Control	6	144.3 ± 99.5	155.0 ± 98.5	10.7 ± 23.4	0.316	0.902
	WB-EMS	9	183.0 ± 89.1	196.6 ± 85.1	13.6 ± 52.2	0.458	
Erythrocytes [×10^6^/μl]	Control	6	3.96 ± 0.65	4.30 ± 0.75	0.34 ± 0.74	0.311	0.564
	WB-EMS	9	3.79 ± 0.50	3.93 ± 0.44	0.15 ± 0.32	0.206	
Hemoglobin [g/dl]	Control	6	12.1 ± 1.4	13.3 ± 2.2	1.1 ± 2.3	0.374	0.391
	WB-EMS	9	12.1 ± 1.2	12.5 ± 1.0	0.4 ± 0.9	0.225	
Hematocrit [%]	Control	6	36.1 ± 4.1	39.0 ± 6.0	2.9 ± 6.4	0.312	0.422
	WB-EMS	9	35.5 ± 3.8	36.5 ± 2.9	1.0 ± 2.7	0.325	
**Clinical chemistry**							
CRP [mg/l]	Control	6	3.7 ± 4.1	6.1 ± 12.3	2.4 ± 9.3	0.600	0.388
	WB-EMS	9	5.7 ± 5.1	5.2 ± 9.0	–0.5 ± 10.3	0.892	
Albumin [g/l]	Control	6	40.3 ± 3.3	41.2 ± 4.0	0.9 ± 4.9	0.676	0.896
	WB-EMS	8	41.0 ± 3.9	42.2 ± 2.5	1.2 ± 3.0	0.303	
Total protein [g/l]	Control	6	62.2 ± 6.0	64.0 ± 7.6	1.8 ± 6.2	0.510	0.382
	WB-EMS	8	65.5 ± 7.7	64.4 ± 5.2	–1.1 ± 5.7	0.602	
Triglycerides [mg/dl]	Control	6	130.5 ± 49.6	166.8 ± 58.8	36.3 ± 50.6	0.139	0.064
	WB-EMS	8	179.0 ± 54.5	147.3 ± 46.3	–31.8 ± 68.7	0.232	
LDH [IU/l]	Control	6	183.0 ± 97.2	187.0 ± 48.1	4.0 ± 92.0	0.919	0.657
	WB-EMS	9	289.0 ± 112.2	269.7 ± 66.3	–19.3 ± 100.4	0.579	
Creatinine [mg/dl]	Control	6	0.91 ± 0.19	0.95 ± 0.29	0.04 ± 0.12	0.446	0.916
	WB-EMS	9	0.92 ± 0.23	0.97 ± 0.28	0.05 ± 0.10	0.174	

*^*1*^Includes patients with pre- and post-assessment data. ^*a*^Paired-samples t-test for parametric or Wilcoxon-signed-rank test for non-parametric values for comparison of pre- and post-intervention values. ^*b*^Independent-samples t-test for parametric or Mann–Whitney U-test for non-parametric values for comparison of pre- to post-test changes. Statistical significance is indicated by p < 0.05 and marked in bold type. WB-EMS, whole-body electromyostimulation; CRP, C-reactive protein; LDH, lactate dehydrogenase.*

## Discussion

This study is the first study, by our knowledge, that investigated the impact of a combined physical exercise and nutrition program on patients actively treated for hematological malignancies. We demonstrated that supervised WB-EMS training seems to be a safe and feasible strength training method for this patient group. Our results showed a significantly higher skeletal muscle in the patients additionally trained by WB-EMS compared to the usual care patients during the 12-week study course. This strengthens our primary hypothesis that a combined exercise and nutrition intervention may be more efficient in maintaining muscle mass during the oncological treatment in hematological cancer patients than a solely nutritional support. The dual therapeutic approach also seemed to induce benefits on the physical functioning and metabolic risk factors with regard to the lipid metabolism of the patients.

### Body Composition

Patients suffering from cancer are faced to a broad range of physiological and psychological symptoms resulting in a decline of physical activity that detrimentally affects body composition and functional status. The causes are multifactorial and include disease- and treatment-related toxicities on the cardiopulmonary, gastrointestinal, neurological and hematopoietic system. As a consequence of inflammation-mediated metabolic changes, increased catabolic processes trigger muscle protein breakdown as a hallmark of cancer cachexia that is mostly prominent in patients with solid tumors (Fearon et al., 2013). In hematological cancer, cytotoxic effects of chemotherapeutic agents, radiation, and immunosuppressive agents may be the main contributors to the prognostic process of muscle wasting (Blauwhoff-Buskermolen et al., 2016; Guglielmi et al., 2017). Synthetic glucocorticoids are widely used within the chemotherapeutic treatment of lymphoproliferative diseases (Lin and Wang, 2016). Disadvantageously, glucocorticoids induce a broad range of catabolic actions and dysregulate anabolic signaling leading to a loss in skeletal muscle mass and function (Gupta and Gupta, 2013). Deficits in health-related physical fitness are therefore common (Persoon et al., 2017b). Increasing the physical activity of cancer patients during active treatment could be a key approach to overcome those deficits and preserve muscle mass. We here provided an novel strength training method in form of WB-EMS that is time-efficient, gentle for the patient and was already shown to increase muscle mass in sarcopenic elderly and patients suffering from chronic heart failure (Fritzsche et al., 2010; Kemmler et al., 2014). With the present pilot trial we could emphasize the muscle-building effect of the WB-EMS training for patients actively treated for different hematological malignancies. Our results demonstrated that an additional regular WB-EMS training can improve skeletal muscle mass with a superior effect to a sole nutritional support even after 4 weeks of study intervention. The benefit of this combined 12-week intervention on muscle mass maintenance is in line with the results of our previous study in advanced-stage solid cancer patients (Schink et al., 2018). Studies evaluating the effect of physical exercise on skeletal muscle mass as a primary outcome in hematological cancer patients during active treatment are quite rare. Evidence of the effectiveness of physical exercise to improve skeletal muscle mass in hematological cancer was provided by a study of Coleman et al. (2003), demonstrating a higher muscle mass for multiple myeloma patients after a 12-week aerobic/resistance exercise program compared to a control group who only received advices for an active lifestyle (Coleman et al., 2003). In contrast, a mixed exercise program consisting of home-based, gym-based and group-based exercises showed no effect on fat-free mass index in myeloma patients (Groeneveldt et al., 2013). Likewise, resistance exercise three or five-times a week showed no superior impact on arm muscle area compared to control patients in acute leukemia patients undergoing bone marrow transplantation and total parenteral nutrition (Cunningham et al., 1986). Overall, it would be of great interest to prospectively investigate, if WB-EMS combined with the high protein nutritional support may be more efficient in improving the muscle mass status than conventional strength training methods.

In fact, we observed a strong positive correlation of the amount of protein intake with an increase in skeletal muscle mass after 12 weeks in the WB-EMS group. This observation may suggest that the known hypertrophic effect of physical exercise, especially in form of resistance training (Schoenfeld, 2010), can be supported and probably enhanced by the additional anabolic stimuli of a regular high protein/amino acid intake that helps to efficiently overcome increased catabolic processes (Willoughby et al., 2007). Tieland et al. (2012) demonstrated that an additional protein supplementation of 15 g 2×/day showed a stronger effect on muscle mass gain in older men performing a progressive resistance training for 24 weeks compared to the exercising placebo-group. Contrary, no effect of an additional protein supplementation (40 g/d) on muscle mass gain during a resistance training program was observed in a study with breast cancer survivors (Madzima et al., 2017). In this study, however, patients reached only a mean daily protein intake of 1.2 g/kg/d in contrast to a mean daily protein intake of 1.4 g/kg/d in our trial and calorie restriction resulted in a net gain of the protein intake of only 17 g/d. This may have affected results. However, due to the small sample size and the lack of an exercise group that was not intended to increase protein intake, a definitive conclusion about a valid impact of the amount of protein intake on muscle build up in our patient population cannot be drawn. Nonetheless, although our correlation analysis should be interpreted cautiously, it emphasizes a further investigation of the exact amount of dietary protein that is needed for muscle build-up in lager-scaled randomized controlled trials.

In addition to skeletal muscle mass, we also monitored changes in bodyweight and other body compartments. At baseline, approximately 60% of our patients were classified as overweight or obese. Unfavorable body composition characterized by overweight/obesity as a result of a high body fat percentage and reduced muscle mass is frequently observed in patients treated for hematological cancer and hints toward the presence of the so called sarcopenic obesity (Xiao et al., 2016; Persoon et al., 2017b). High body fat promotes the secretion of inflammatory mediators by the adipose tissue leading to a systemic inflammation and higher oxidative stress level that trigger the development of metabolic diseases (Nanayakkara et al., 2012; Ellulu et al., 2017). Thus, sarcopenic obesity can negatively impact the overall life expectancy (Yip et al., 2015). Considering this, avoiding deterioration of manifestation of metabolic disorders of long-term cancer patients should be goal in a multimodal cancer care. Studies also provide evidence that high body fat can be predictive for greater dose-independent side-effects and a worse treatment response (GroDelta et al., 2017). Our results showed that even though both study groups tended to increase in bodyweight, this increase was due different tissues changes. While patients of the WB-EMS group increased in muscle mass and were balanced in body fat percentage, control patients were further shifted toward sarcopenic obesity, underlined by a decrease in muscle mass and an increase in body fat that was significantly higher at study end. Interestingly, a trial of Kemmler et al. (2017) demonstrated comparable results. Older men receiving WB-EMS training and high protein supplementation (1.7–1.8 g/kg/d) showed a higher reduction of total body fat and increase of muscle mass than men with isolated protein supplementation or no intervention. The increase in body fat during treatment of hematological cancer might be a result of glucocorticoid treatment affecting the lipid metabolism (Peckett et al., 2011) in line with low physical activity levels induced by fatigue (Coleman et al., 2011). Our results therefore suggest that exercise in form of WB-EMS combined with a high protein intake could be efficient to attenuate treatment-related unfavorable body composition changes and may therefore also be helpful to improve treatment response and avoid metabolic diseases in the post-treatment period. Underlining this thesis, we observed changes in serum triglycerides. The lipid profile of patients with hematological malignancies was shown to be negatively altered. Concentrations in cholesterol fractions are suggested to be inversely associated with the incidence of hematological cancer, while triglycerides can be elevated in those patients, especially in the active disease period (Naik et al., 2006; Kuliszkiewicz-Janus et al., 2008). After 12 weeks we recognized a strong trend toward a decrease in serum triglycerides in the WB-EMS group in contrast to an increase in the control group.

During the study course BIA measurements revealed no statistically significant differences in the nutritional and health-predicting parameter of phase angle (Lee et al., 2014). Different glucocorticoid treatments and antidiuretic drugs may have probably influenced body water distribution that highly affects phase angle values.

We assessed body composition and thus skeletal muscle mass by BIA as it was demonstrated to correlate with the results obtained from Dual Energy X-ray Absorptiometry and magnetic resonance tomography (Bosy-Westphal et al., 2008; Bosy-Westphal and Jensen, 2017), but is much easier and faster to apply, as previously described (Schink et al., 2018).

### Physical Function

Functional disabilities after treatment for hematological cancer are common and can be observed even years after the initial therapy (Battaglini, 2011; Tuchman et al., 2015). Several reviews suggest that muscle strength and physical functioning can be improved by different exercise interventions in these patients (Bergenthal et al., 2014; Gan et al., 2016). Our results support these findings. A significantly higher 6-min-walking distance and improved patient-reported performance status (Karnofsky-Index) suggest the effectiveness of WB-EMS to stabilize and ameliorate the whole muscular status of the patients by improving functional capacity. In a study with various hematological cancer patients an exercise program did not stabilize 6-min-walking distance during HSCT (Takekiyo et al., 2015), even though exercise was conducted 5 days a week each time for 40 min. In contrast, Mello et al. (2003) showed a significant increase in muscle strength 6 weeks after allogenic bone marrow transplantation. So, either scheduling of exercise programs or the appropriate intensity and application type of exercise may be important to relevantly improve physical function in patients with hematological malignancies. Compared to the conventional exercise programs the less time-consuming exercise schedule of our ambulant WB-EMS training program (2×/week á 20 min) may offer a great advantage to efficiently sustain physical function in hematological patients even during active treatment.

### Quality of Life

Maintaining of the quality of life is an important goal in a multimodal cancer care concept. A review of Allart-Vorelli et al. (2015) demonstrated that different dimensions of quality of life including physical and psycho-social well-being are detrimentally affected by hematological malignancies and treatment side-effects. Fatigue is thereby one of the most burdening symptoms in these patients (Miltenyi et al., 2010). Physical exercise was shown to improve physical well-being and to potentially reduce fatigue-symptoms (Oldervoll et al., 2003; Groeneveldt et al., 2013). Both of our study groups improved in fatigue, whereby a greater improvement in the control group was most likely due to a higher fatigue burden at baseline of the analyzed patients. However, a recent study with myeloma and lymphoma patients also showed a decrease in fatigue in the exercising as well as in the usual care control group, probably a result of exercise contamination in controls (Persoon et al., 2017a). Besides physical exercise, also the nutritional support covering patients’ energy and nutrient requirements may have relieved fatigue symptoms. The small sample size makes it difficult to interpret the results conclusively. However, although there are no significant differences between the groups, patients of the physical exercise group showed greater improvements for their psychosocial functioning, indicating improved social and role functioning. These patients showed also a distinct increase in global health/overall quality of life after 12 weeks of WB-EMS training. Moreover, intra-group analysis revealed a significant amelioration of insomnia, a frequent symptom in hematological cancer patients (Johnsen et al., 2009; Coleman et al., 2011). Exercise was previously suggested to be a potent treatment against insomnia (Passos et al., 2012). Further, a strong trend toward decreased nausea during anti-cancer treatment was revealed for the WB-EMS group that could also be demonstrated in exercising breast cancer patients undergoing adjuvant chemotherapy (Lee et al., 2008) and hints toward a decreased treatment-related toxicity and adequately adapted nutrition.

### Overall Training Adherence

The implementation of exercise interventions into the clinical routine, especially for actively treated hematological cancer patients, is not easy. Many exercise studies were conducted with solid cancer patients, especially breast and prostate cancer patients that are relatively fit. However, treatment toxicities including anemia-induced fatigue, insomnia, and psychological distress hinders hematologic patients from regular exercise (Coleman et al., 2003). The high dropout rate in our physical exercise group of 59.1% underlines this difficulty. Deterioration in clinical status and treatment toxicities (worsening of acute GvHD) were the main reasons for study dropouts. Poor state of health was underlined by the significantly higher daily drug intake and leukocyte count as well as lower serum albumin hinting toward decreased nutritional status. Only visiting the monthly intermediate measurement may have been therefore less burdening for patients than regularly attending the WB-EMS exercise sessions that could interfere with disease-related complications (Oldervoll et al., 2011; Schink et al., 2018). Nonetheless, patients who completed the 12-week intervention period of our study showed a good exercise adherence attaining in mean 77.3% of the scheduled exercise sessions underlining the good acceptance of the WB-EMS training method. Moreover, it has to be mentioned that no WB-EMS related adverse events occurred and that approximately one-third of our patients suffered from osteolytic lesions, as a complication of myeloma infiltration. Those patients might not have been able to undergo a strenuous high intensity apparatus training that is necessary to build up muscles but can be too risky due to possible bone fractures. The innovative technology of WB-EMS to activate almost all large muscle groups simultaneously in line with gentle, easy-to-perform mild exercises removes those concerns.

### Study Limitations

Even though, baseline characteristics were well balanced between study groups, the results may be limited by the lack of randomization and blinding of study patients and assessors, respectively, as well as by the small sample size that could have induced bias (Schink et al., 2018). Outcome differences between the study groups may have been a result of a higher percentage of more motivated patients in the WB-EMS group, albeit allocation to study groups was associated to the journey way to the study center to mostly rule out this problem (Schink et al., 2018). Hence, randomized controlled trials are now necessary to confirm our promising results.

## Conclusion

Summarizing the results of our pilot study we could demonstrate that physical exercise in form of WB-EMS seems to be a feasible and safe strength training method for patients with hematological malignancies. In combination with individual nutritional support high in dietary protein WB-EMS potentially improves skeletal muscle mass and prevents treatment- and disease-related unfavorable changes in body composition and fat metabolism. The clinical relevance of our findings may be emphasized by the observed improvement and preservation of the physical function by the combined therapeutic approach. Our preliminary results now encourage the conduction of further randomized controlled trials with longer follow-up periods to verify these findings and to investigate the maintenance of muscle mass und function after the active treatment period of hematological cancer patients.

## Data Availability Statement

The raw data supporting the conclusions of this manuscript will be made available by the authors, without undue reservation, to any qualified researcher.

## Author Contributions

KS, HH, and YZ substantially contributed to the study conception, design, and conduction. KS was responsible for the data acquisition and wrote the manuscript. KS and JM analyzed the data. HH, DR, AM, MN, and YZ revised the manuscript critically for important intellectual content. KS and YZ had primary responsibility for final content. All authors read and approved the final manuscript.

## Conflict of Interest Statement

The authors declare that the research was conducted in the absence of any commercial or financial relationships that could be construed as a potential conflict of interest.
